# Catabolite control protein C contributes to virulence and hydrogen peroxide-induced oxidative stress responses in *Listeria monocytogenes*

**DOI:** 10.3389/fmicb.2024.1403694

**Published:** 2024-05-31

**Authors:** Seto C. Ogunleye, Shamima Islam, Q. M. Monzur Kader Chowdhury, Ozan Ozdemir, Mark L. Lawrence, Hossam Abdelhamed

**Affiliations:** Department of Comparative Biomedical Sciences, College of Veterinary Medicine, Mississippi State University, Mississippi, MS, United States

**Keywords:** *Listeria*, oxidative stress, RNA-seq, virulence factor, biofilm

## Abstract

*Listeria monocytogenes* causes listeriosis, an infectious and potentially fatal disease of animals and humans. A diverse network of transcriptional regulators, including LysR-type catabolite control protein C (CcpC), is critical for the survival of *L. monocytogenes* and its ability to transition into the host environment. In this study, we explored the physiological and genetic consequences of deleting *ccpC* and the effects of such deletion on the ability of *L. monocytogenes* to cause disease. We found that *ccpC* deletion did not impact hemolytic activity, whereas it resulted in significant reductions in phospholipase activities. Western blotting revealed that the Δ*ccpC* strain produced significantly reduced levels of the cholesterol-dependent cytolysin LLO relative to the wildtype F2365 strain. However, the Δ*ccpC* mutant displayed no significant intracellular growth defect in macrophages. Furthermore, Δ*ccpC* strain exhibited reduction in plaque numbers in fibroblasts compared to F2365, but plaque size was not significantly affected by *ccpC* deletion. In a murine model system, the Δ*ccpC* strain exhibited a significantly reduced bacterial burden in the liver and spleen compared to the wildtype F2365 strain. Interestingly, the deletion of this gene also enhanced the survival of *L. monocytogenes* under conditions of H_2_O_2_-induced oxidative stress. Transcriptomic analyses performed under H_2_O_2_-induced oxidative stress conditions revealed that DNA repair, cellular responses to DNA damage and stress, metalloregulatory proteins, and genes involved in the biosynthesis of peptidoglycan and teichoic acids were significantly induced in the *ccpC* deletion strain relative to F2365. In contrast, genes encoding internalin, 1-phosphatidylinositol phosphodiesterase, and genes associated with sugar-specific phosphotransferase system components, porphyrin, branched-chain amino acids, and pentose phosphate pathway were significantly downregulated in the *ccpC* deletion strain relative to F2365. This finding highlights CcpC as a key factor that regulates *L. monocytogenes* physiology and responses to oxidative stress by controlling the expression of important metabolic pathways.

## 1 Introduction

*Listeria monocytogenes* is a foodborne pathogen responsible for listeriosis, which is characterized by high hospitalization and fatality rates (Drevets and Bronze, 2008; Eallonardo et al., 2023). In healthy individuals, listeriosis generally manifests in the form of noninvasive gastroenteritis, whereas immunocompromised individuals can experience severe outcomes including septicemia, abortion, and neurological disorders such as meningoencephalitis (Buchanan et al., 2017). The ability of *L. monocytogenes* to cause diseases is attributable to a coordinated series of virulence activities mostly regulated by the pleiotropic transcriptional activator, PrfA (Positive Regulatory Factor A) (Lampidis et al., 1994). PrfA serves as a master virulence factor responsible for controlling the transcription of several virulence factors, including phosphatidylinositol-specific phospholipase C (*plcA*), the cholesterol-dependent cytolysin LLO (*hly*), the zinc metalloproteinase Mpl (*mpl*), the actin assembly-inducing protein ActA (*actA*), phosphatidylcholine phospholipase C (*plcB*), and internalins A and B (*inlA* and *inlB*). These gene clusters are integral for host cell invasion, intracellular growth and replication, and the cell-to-cell spread of *L. monocytogenes* (Camejo et al., 2011; Kanki et al., 2018; Wiktorczyk-Kapischke et al., 2023).

*Listeria monocytogenes* is considered a ubiquitous microorganism that can adapt, survive, and even grow in a wide variety of habitats under various environmental stress conditions (Gray et al., 2021; Lakicevic et al., 2022; Osek et al., 2022). A large network of complex transcriptional regulators enables *L. monocytogenes* to rapidly respond and adapt to diverse settings including extracellular, abiotic, and intracellular environments (Xayarath and Freitag, 2012). Among these networks of regulators, LysR-type transcriptional regulators (LTTRs) have been reported to play roles in regulating gene expression in response to environmental changes and various stressors (Reniere et al., 2015; Biswas et al., 2020). LTTRs control genes involved in cellular metabolism, pathogen virulence, cell wall production, flagellar attachment/modification, pathogen motility, quorum sensing, stress responses, and toxin production and secretion (Cao et al., 2001; Russell et al., 2004; Maddocks and Oyston, 2008; Zhang et al., 2018).

The catabolite control protein C (CcpC) is an uncharacterized LTTR in *L. monocytogenes*, encoded by the *ccpC* gene (Mittal et al., 2013). The *ccpC* gene is flanked upstream by *cbpB* gene encoding a protein with tandem cystathionine-β-synthase (CBS) domains that binds c-di-AMP and contributes to solute importation (Kim et al., 2002; Huynh and Woodward, 2016). Downstream, *ccpC* is flanked by the *dapD* gene, which encodes a protein involved in diaminopimelate and lysine biosynthesis (Kim et al., 2006). In *L. monocytogenes*, CcpC plays an integral role in controlling genes encoding enzymes involved in the tricarboxylic acid (TCA) cycle including citrate synthase (*citZ*), aconitase (*citB*), and isocitrate dehydrogenase (*citC*) in response to the intracellular concentration of citrate (Kim et al., 2006; Mittal et al., 2013). The promoter regions of the *citZ* and *citB* genes are potential binding sites for CcpC, leading to the disruption of citrate synthesis by preventing read-through transcription (Mittal et al., 2013). The presence of citrate inhibits the interaction of CcpC with these promoter regions (Kim et al., 2002; Pechter et al., 2013). While the role that CcpC plays in metabolism and the regulation of the TCA cycle is known, information on the contributions of CcpC to pathogenesis is limited. The goal of this study was thus to investigate the role of CcpC in virulence and intracellular survival of *L. monocytogenes*, to evaluate the impact of CcpC deletion on biofilm formation and survival of *L. monocytogenes* under different stress conditions, and to identify associated regulons.

## 2 Materials and methods

### 2.1 Bacterial strains and growth conditions

*Listeria monocytogenes* F2365 4b strains were grown in brain heart infusion (BHI) broth and agar (Difco) at 37°C. *Escherichia coli* DH5α strain was grown in Luria-Bertani (LB) (Difco Laboratories) broth and agar (Table 1). Macrophage J774A and fibroblast (CRL-2648; ATCC) cell lines were grown in Dulbecco’s modified Eagle’s medium (DMEM) (ATCC, Manassas, VA) supplemented with 10% fetal bovine serum (FBS) (Atlanta Biologicals, Norcross, GA) and 1% glutamine. Cultures were maintained at 37°C with 5% CO_2_ under humidified conditions. Furthermore, BHI broth supplemented with 1% glucose (Sigma-Aldrich) was used to determine biofilm formation. Antibiotics such as erythromycin (20 μg/mL), ampicillin (50 μg/mL), kanamycin (50 μg/mL), and neomycin (20 μg/mL) were used for mutant and complemented strain construction while gentamicin (20 μg/mL) was used for cell line culturing. Brilliance *Listeria* agar (BLA) supplemented with lecithin (Oxoid) was used to detect phospholipase activity of bacterial strains.

**Table 1:** Bacterial strains, plasmids, and primers used in this study.

Bacterial strain, plasmid, or primer	Description or sequence	Source or reference
**Bacterial strains**		
*E. coli* DH5α	Competent cells	Standard laboratory strain
***L. monocytogenes***		
F2365	Wildtype serotype 4b strain	Nelson et al. (2004)
F2365Δ*ccpC*	F2365Δ*ccpC* mutant strain	This study
F2365Δ*ccpC::p*PL2-*ccpC*	F2365Δ*ccpC::p*PL2-*ccpC* complement strain	This study
F2365Δ*hly*	F2365Δ*hly* mutant strain	Portnoy lab
F2365Δ*plcA*	F2365Δ*plcA* mutant strain	Portnoy lab
**Plasmid**		
pHoss1	8,995 bp, pMAD,::*secY* antisense, *ΔbgaB*, Amp^r^, Ery^r^	Abdelhamed et al. (2015)
*p*PL2	6,123 bp, PSA *attPP*, Chl^r^	Lauer et al. (2002)
P*ccpC*plasmid	pHoss,::Δ*ccpC*, Amp^r^, Ery^r^	Abdelhamed et al. (2015)
*p*PL2-ccpC	*p*PL2,::Δ*ccpC*, Amp^r^, Ery^r^	This study
**Primers used for construction Δ*ccpC* strain (5′-3′)**		
ccpC-A	aaaggg**gaattc**CCACGCGCGTATAATAGCAA	EcoRI
ccpC-B	TTTCCTCCACCTGCAAATGA	
ccpC-C	TCATTTGCAGGTGGAGGAAAAACAGAACGTATCGGAAGGG	
ccpC-D	aaaggg**gtcgac**CAAAATCCCCGTACGCCATT	SalI
ccpC-Seq	GCAAACGGATTTCCCAGTAA	
**Primers used for construction complemented strain**		
ccpC-CompF1	aaattc**gagctc**GCAGGTGGAGGAAAATAGATG	SacI
ccpC-CompR1	aaattc**gtcgac**ATTTTGCCATAGCTATTTAACTTGTTC	SalI
**Primers for RT-qPCR (5′-3′)**		
plcA-F	GCATCACTTTCAGGCGTATTAG	
plcA-R	CGTGTCAGTTCTGGGAGTAG	
plcB- F	GCTTGACCGCAAGTGTTCTA	
plcB- R	GATTATCCGCGGACCAACTAAG	
gutM-F	GTTGCGGATAAATATGCTGAGAAA	
gutM-R	TGATGATAGTTGGTGAAAGTCTTGA	
inlB-F	GATGCGCTTCCTGCTTTAGA	
inlB-R	GAAAGTCCAGCATCCTCCATATT	
inlA_F	CGGCAAAGAAACAACCAAAGA	
inlA_R	GCATCAAACCACCCAACAAA	
recA-F	AGGCGAGCTTGTTGATATGG	
recA-R	CTTCCGTCGATTTCGTACTCTT	
lexA-F	GAAGCAGAGACACCCAATGT	
lexA-R	TCATACTTTCGCCGTCGATTT	
dnaA-F	CCTAGTTACGACACATGGATGAA	
dnaA-R	TCCTGCTCGCCATCAATAAA	
lspA-F	TGCCAAAGGAAAGCGACTAT	
lspA-R	GAGCATTAGTACGACACCAACT	
fur-F	CGGTGTTTCTCGGTATGACTT	
fur-R	GTCTGCAATCTGTGCAAATCC	
16S rRNA-F	CAAGCGTTGTCCGGATTTATTG	
16S rRNA-R	GCACTCCAGTCTTCCAGTTT	
LLO-F	CAAATGTGCCGCCAAGAAA	
LLO-R	CGAGAGCACCTGGATATGTTAG	
Crp/fnr-F	TAGGCGCAACCAACAGATT	
Crp/fnr-R	GTAAGCGGCCCGATACATT	
actA-F	GAAACAGCACCTTCGCTAGA	
actA-R	CTCTCCCGTTCAACTCTTCTTC	

Underlined text denotes primer overlap with the ccpC-B primer, and bold faces in the primer sequences above indicate restriction enzyme sites. Amp^r^, ampicillin resistant; Ery^r^, erythromycin resistant; Chl^r^, chloramphenicol resistant.

### 2.2 Construction of *∆ccpC* and complemented strains

The catabolite control protein C (*ccpC*) gene was targeted for in-frame deletion from *L. monocytogenes* F2365 using an allelic exchange technique as previously described (Abdelhamed et al., 2015). Four primers (A, B, C, and D) were designed for amplification of upstream (A and B) and downstream (C and D) regions of the *ccpC* gene using PCR (Table 1). The upstream and downstream amplicons were ligated through overlap extension PCR using A and D primers. This was followed by cloning into pHoss1 plasmid using *E. coli* DH5α. The resulting plasmid was transformed into F2365 by electroporation for integration by homologous recombination. The *ccpC* deletion was confirmed by PCR and sequencing. A complementation strain was made by amplifying a DNA fragment containing the entire *ccpC* gene and its promoter from the F2365 genome and ligating it into pPL2 shuttle integration vector (Lauer et al., 2002). The resulting plasmid was electroporated into *∆ccpC* to obtain the complemented strain designated as F2365Δ*ccpC*::pPL2-*ccpC* (C∆*ccpC*) (Table 1).

### 2.3 Hemolytic activity assays

The hemolytic activity of F2365, Δ*ccpC*, and C∆*ccpC* strains was determined as previously described (Alonzo et al., 2009). Briefly, overnight cultures of bacterial strains were diluted in 1:10 in BHI broth and grown at 37°C for 4 h to an OD_600_ of approximately 0.7. Bacterial supernatants were obtained by centrifugation, and 500 μL of 1% sheep RBC (in activation buffer) was added and incubated at 37°C for 1 h. After incubation, the supernatant was collected by centrifugation and transferred into 48 well plates. Hemolytic activities were quantified by measuring the absorbance at OD_450_ nm with SpectraMax M5 ELISA reader (Molecular Devices, Sunnyvale, CA, United States). All experiments were performed three times independently with three replicates in each.

### 2.4 Phospholipase activity assay

Phospholipase activity of F2365, Δ*ccpC*, and C∆*ccpC* strains was tested using BLA supplemented with lecithin (Oxoid) as previously described (Blank et al., 2014). Briefly, bacteria were streaked onto the BLA and incubated for 48 h at 37°C, followed by measurement of the zone of opacity surrounding the bacterial growth. All experiments were performed three independent times with three replicates each.

### 2.5 Detection of Listeriolysin O protein levels in *Listeria monocytogenes*

The amount of LLO protein present in Δ*ccpC* was compared to F2365 and C∆*ccpC* strains using Western blot with LLO polyclonal antibodies. Protein from samples was extracted as previously described, with modifications (Alonzo et al., 2009). Bacterial pellets were obtained by centrifugation and lysed with cell lysis buffer (50 mM Tris–HCl pH 8.0, 5% glycerol, 0.5% triton X-100, 2 mM PMSF, and 1.5 mM EDTA) at 4°C for 30 min. Sonication was performed shortly before addition of 20 μL/mL DNase followed by incubation at 4°C for 1 h, and supernatant was obtained by centrifugation. The protein was suspended into 4X Laemmli sample buffer containing β-mercaptoethanol. The samples were heated for 10 min at 100°C, followed by Western blotting. LLO (primary antibody) and HRP-conjugated goat anti-rabbit antibody were used to detect LLO expression in the protein extracts. P-60 antibody (primary antibody) and HRP-conjugated anti-mouse antibody (secondary antibody) was used for P60 expression as the control (Bubert et al., 1997).

### 2.6 Biofilm formation

Biofilm formation by Δ*ccpC* and C∆*ccpC* was compared to the wildtype F2365 by static growth after crystal violet staining as described previously (Wakimoto et al., 2004). Briefly, overnight cultures were diluted 100-fold in BHI broth supplemented with 1% glucose (Sigma-Aldrich) and incubated in 48-well plates under static condition for 24, 48 and 72 h, including negative control. At the indicated time points, wells were gently washed with PBS, and adherent cells were stained with 0.1% crystal violet (Sigma-Aldrich) for 10 min at room temperature. Plates were rinsed with PBS, and the residual crystal violet was solubilized with 70% ethanol. Biofilm formation was quantified by measuring absorbance at 538 nm with a SpectraMax M5 ELISA reader (Molecular Devices, Sunnyvale, CA, United States). Biofilm formation was determined three independent times with eight replicates each.

### 2.7 Intracellular replication

Intracellular replication in macrophages by Δ*ccpC* was compared to F2365 and C∆*ccpC* using a method previously described (Vargas García et al., 2015). Macrophages were seeded in 48-well tissue culture plates and confluent monolayers were infected with bacterial suspension in phosphate buffered solution (PBS) at multiplicity of infection (MOI) of approximately 1 to 10. After 1 h incubation, the cells were washed with PBS and incubated in DMEM containing a low dose of gentamicin (20 μg/mL) to kill extracellular bacteria. After 4 h incubation, cells were washed with PBS, lysed, and the released bacteria were resuspended before being plated on BHI agar. Bacterial colonies were counted, and CFU/mL calculated (log_10_). All infections were performed three independent times, and four replicates were performed for each infection.

### 2.8 Plaque formation

Plaque formation by Δ*ccpC* was compared to F2365 and C∆*ccpC* using murine L2 fibroblast cells as described previously (Jones and Portnoy, 1994). Fibroblast monolayers were grown in 75 cm^2^ plastic flasks (Sigma-Aldrich) at 37°C under 5% CO_2_. Cells were seeded at a concentration of 10^6^ cells/well in a 6-well tissue culture plate to form a confluent monolayer before inoculation with *L. monocytogenes* strains. After incubation for 1 h, DMEM containing 20 ug/mL gentamicin was added, and plates were incubated at 37°C and 5% CO_2_ for 4 days. Living cells were visualized by adding an additional overlay consisting of DMEM, 0.5% agarose, and 0.1% neutral red and incubated overnight. Plaque sizes and numbers were determined using a compound microscope with ImageJ, and scores were converted to percentages. This experiment was done three independent times with at least 3 replicates.

### 2.9 Quantitative real-time PCR analysis of gene transcription

The expression of six virulence genes, namely *plcA*, *plcB*, *inlA*, *inlB*, *actA*, and *hly*, was compared in wildtype F2365 and Δ*ccpC* strains during growth in BHI. The wildtype F2365 and Δ*ccpC* strains were grown in BHI broth overnight at 37°C and bacterial pellets were obtained by centrifugation at 15,000 × g for 10 min at 4°C. Total RNA was extracted using the FastRNA spin kit for microbes and the FastPrep-24 instrument (MP Biomedicals, Santa Ana, CA) by following the manufacturer’s instructions. For each strain, total RNA was isolated from 3 independent biological replicates. Genomic DNA was eliminated from the total RNA by using on-column DNase treatment with an RNase-free DNase set (Qiagen, Hilden, Germany). The quantity and quality of total RNA were analyzed using a NanoDrop ND-1000 spectrophotometer (Thermo Scientific, United States) by measuring the OD_260_/OD_280_ ratio. Extracted RNA was transcribed into complementary DNA (cDNA) by reverse transcriptase from total RNA, and the cDNA was then used as the template for the RT-qPCR. Primers were designed with IDT (Integrated DNA Technologies) software. The product of the first-strand cDNA synthesis was diluted 50 times before use. RT-qPCR was performed in a 20- μl reaction volume containing 5 μL of cDNA, 10 μL of SYBR green real-time PCR master mix (Roche Diagnostic GmbH, Mannheim, Germany), 0.6 μL of gene-specific primers (10 μM), and 3.8 μL water. Amplification and detection of specific products were performed with the Mx3000P real-time PCR system (Stratagene) with the following cycle profile: initial denaturation at 95°C for 10 min, 40 cycles of 95°C for 30 s, 55°C for 30 s, and 72°C for 1 min. The expression of each gene was normalized against the expression of the housekeeping gene, 16S rRNA, before comparative analysis. Further, DNA melting curve analysis at the end of each run ensured that the desired amplicon was detected and that no secondary products were amplified. For each gene, triplicate assays were done. Expression levels of the tested genes were quantified by the relative quantitative method (2^−^∆∆CT^). RT-qPCR was performed to validate 13 differentially expressed genes from RNA-seq data. Primers and gene information are listed in Table 1. RT-qPCR was performed on the same RNAs used for RNA-seq. cDNA synthesis and RT-qPCR were performed as described above.

### 2.10 Scanning electron microscopy

Scanning electron microscopy (SEM) was performed to observe biofilm formation following overnight growth in BHI at 37°C. The biofilm formation of *L. monocytogenes* Δ*ccpC* and F2365 was studied with three replicates. Briefly, bacterial culture was gently centrifuged, washed with PBS, and fixed with fixative (2% paraformaldehyde, 2.5% glutaraldehyde, and 2 mM CaCl_2_ in 0.1 M sodium cacodylate buffer pH 7.4) for 2 h at room temperature. The samples were then washed twice in sodium-cacodylate buffer (0.1 M, pH 7.4) before post-fixation using 1% osmium tetroxide for 1 h Glass cover slides were placed into a sterile polystyrene 6 well plate. Then 1:10 suspension of poly L- Lysine was used to coat the cover slide. After drying and sterilization of the coated glass slide with UV light, aliquots of 2 mL bacterial suspension were inoculated in each well and incubated for 24 h. The non-adherent bacteria were removed by washing with sterile water. Then the adherent bacteria were transferred into a graded mixture of hexamethyldisilazane (HMDS) and ethanol (30, 50, 70, 80, 85, 95 and 100%), followed by a second 100% dehydration for 1 h. This was followed by overnight air-drying, mounting on metal stub using two-sided carbon sticky tape, coating by 45 nm of platinum in EMS Coater operations, and finally examining by SEM.

### 2.11 *In vivo* virulence in Swiss Webster mice

Approval was obtained from the Institutional Animal Care and Use Committee (IACUC) for animal procedures (18-508), and experiments were conducted at the College of Veterinary Medicine. Swiss Webster mice were obtained from Charles River laboratories and housed at 5 mice per cage. Virulence of the Δ*ccpC* and C∆*ccpC* strains were compared to F2365 and negative controls in this study. Overnight culture of bacterial strains (OD_600_ of approximately 1.00) was diluted to a final concentration of 2 × 10^4^ colony forming units (CFU)/mL and injected intravenously through tail vein (Bou Ghanem et al., 2012). At 72 h post-infection, mice were euthanized, and livers and spleens of infected animals were collected, homogenized with saline, and spread on agar plates for CFU determination. All experiments were performed two independent times.

### 2.12 Oxidative stress response

The oxidative stress response of Δ*ccpC* and C∆*ccpC* strains was compared to F2365 using hydrogen peroxide (H_2_O_2_) as the oxidative stressor. Briefly, overnight cultures of bacterial strains were diluted 1:10 in BHI and incubated at 37°C with shaking for ~1 h to reach OD_600_ ~ 0.2–0.3. Pellets were obtained by centrifugation and resuspended in PBS. BHI containing hydrogen peroxide (H_2_O_2_) at 6, 8, and 10 mM were inoculated with the corresponding bacterial strain and used for bacterial enumerations and growth curves. Bacterial enumeration was performed by serially diluting 100 μL samples of the incubated BHI at 1-, 3-, 6-, and 24 h time points and spreading on BHI plates for colony count. Growth curves were conducted using a Cytation 5 Cell Imaging Multi-Mode Reader (BioTek) over 48 h. Oxidative stress response tests were conducted in at least 3 independent experiments with 6 replicates in each experiment.

### 2.13 RNA extraction, library preparation, and transcriptome sequencing

The wildtype F2365 and Δ*ccpC* strains were observed for phenotypic changes under oxidative stress condition and prepared for transcriptomic analysis. Total RNA was extracted from bacterial cultures of F2365 and Δ*ccpC* following exposure to 8 mM H_2_O_2_ for 2.5 h. RNA was extracted as described above in section 2.9. A Ribo-Zero magnetic kit for Gram-positive bacteria (Epicentre) was used to remove rRNAs, and then a fragmentation buffer was added to fragment mRNAs. Before library construction, the concentration of RNA was normalized using specific ScriptSeq kits (Epicentre). Library construction and sequencing was performed by Novogen©. Briefly, mRNA fragments were reverse transcribed to single-stranded cDNAs using random hexamers as primers (Promega). The cDNA libraries were subjected to sequencing using the HiSeq platform (Illumina). For each strain, three independent biological replicates were sequenced.

### 2.14 Sequence mapping, differential expression, and GO and KEGG pathway enrichment analyses

Raw data were filtered to remove reads containing adapters or low-quality reads. The resulting reads were mapped to the genome of *L. monocytogenes* F2365 using Bowtie2. Transcriptomic analysis was conducted using the Bioconductor Edge R analysis package. Transcripts for each sample were quantified and normalized as the number of reads per kilobase per million reads (RPKM). Three replicate RPKM values for each sample were standardized on the basis of their mean transcript values and used to assess gene expression and fold change differences in expression. To minimize false-positive results, a stringent cutoff false discovery rate (FDR) of 1 was applied when identifying differentially expressed genes of the wildtype compared to the mutant strains. GO enrichment analysis was conducted by GOseq (Young et al., 2010), which is based on Wallenius noncentral hypergeometric distribution. GO covers molecular functions, biological processes, and cellular components. The differentially expressed gene list was mapped to the Kyoto Encyclopedia of Genes and Genomes (KEGG) to identify significantly enriched metabolic pathways or signal transduction pathways.

### 2.15 Statistical analysis

Dot plots and median values of bacterial concentrations in each mouse tissue were generated using GraphPad Prism 9.0 software. For *in vivo* experiments, a nonparametric Mann–Whitney test was used to detect statistical significance between F2365, Δ*ccpC*, and C∆*ccpC* treatment groups in bacterial concentrations in liver and spleen of infected mice. Statistical analysis of the hemolytic activity assay, intracellular replication, plaque scores, phospholipase activity, biofilm formation, and oxidative stress were done to compare Δ*ccpC* and wildtype F2365 using Student *t*-test using GraphPad Prism 9.0 software. *p* values of < 0.05 were considered statistically significant in all analyses. ImageJ was used for quantification of bands from Western blot images, and statistical analysis was performed using Student *t*-test.

## 3 Results

### 3.1 CcpC influences the phospholipase activity of *Listeria monocytogenes*

**Figure 1:**
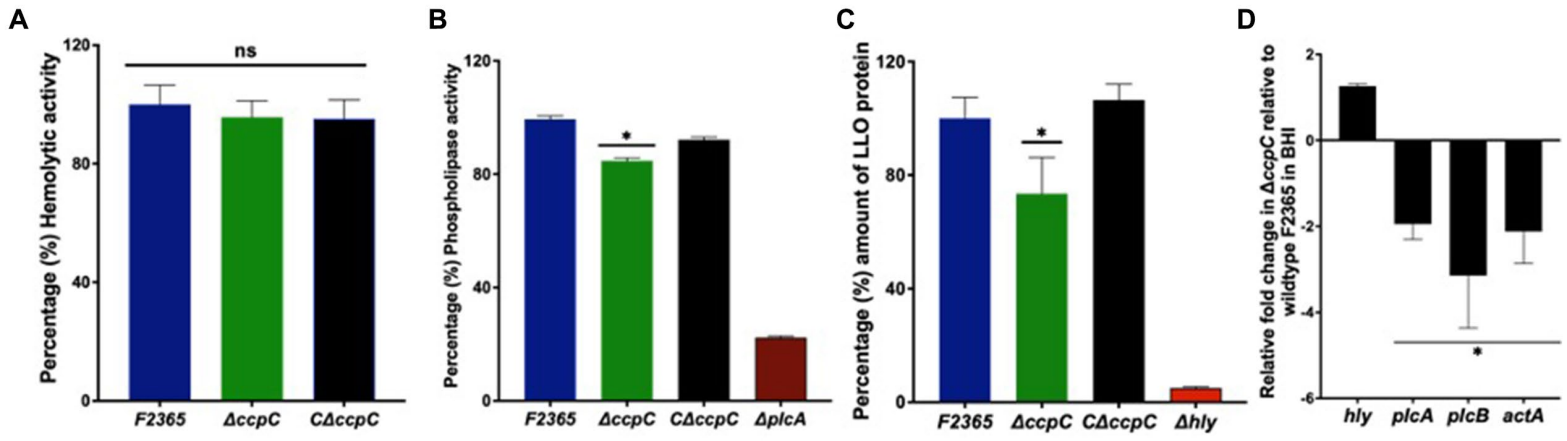
The hemolytic activity, phospholipase activity, and intracellular replication of the Δ*ccpC* and wildtype F2365. **(A)** Percentages of hemolytic activity in sheep erythrocytes by Δ*ccpC* strain showed non-significant ~2% reduction in hemolytic activity compared to the wildtype F2365 (*p* > 0.05). **(B)** The Δ*ccpC* strain exhibited ~20% reduction in phospholipase activity compared to the wildtype F2365 strain when examined on BLA plates containing lecithin (*p* < 0.05). The C∆*ccpC* partially restored the phospholipase activity with opacity zone size similar to F2365. **(C)** The Δ*ccpC* strain on western blot showed a significantly reduced LLO protein level (~25%) compared to the wildtype F2365 (*p* < 0.05), while the C∆*ccpC* restored the amount of LLO protein to the wildtype F2365 level. **(D)** The effect of *ccpC* deletion on the relative expression of *plcA*, *plcB*, *actA*, and *hly* during the growth of *L. monocytogenes* strains in BHI broth using RT-qPCR. The expression of *plcA*, *plcB*, and *actA* genes was significantly downregulated in the Δ*ccpC* strain compared to the wildtype F2365 (*p* < 0.05), whereas *hly* gene was insignificantly upregulated (*p* > 0.05). Bars indicate the standard error of the mean (SEM) of six replicates. The data were compared with Student’s *t*-tests. Asterisks (*) indicate significant differences, *p* < 0.05. NS, No significant difference.

Expression of phospholipase genes *plcA* and *plcB* and the LLO encoded by *hly* gene are controlled by PrfA following its activation in host cells (Moors et al., 1999; Seveau, 2014). To establish whether CcpC impacts PrfA-regulon function, the hemolytic activity of Δ*ccpC* and F2365 was assessed by monitoring the lysis of sheep erythrocytes by bacterial supernatant (Nilsson and Nilsson, 1984). The Δ*ccpC* strain exhibited a non-significant ~2% reduction in hemolytic activity compared to the wildtype F2365 (Figure 1A).

To characterize phospholipase activity, the lecithinase activity of the Δ*ccpC* and wildtype strains was examined on BLA plates containing lecithin. After incubation at 37°C for 72 h, the mean opaque zone size surrounding the ∆*ccpC* colonies was about 20% smaller than that produced by the wildtype strain, indicating reduced secreted phospholipase activity. The C∆*ccpC* exhibited partially restored phospholipase activity with opacity zone size similar to F2365 (Figure 1B). Surprisingly, western blotting showed that LLO protein levels were significantly reduced by approximately 25% in the Δ*ccpC* strain compared to the wildtype F2365 strain (Figure 1C). The LLO protein levels of the complemented C∆*ccp*C strain was similar to wildtype levels. Our results thus revealed a significant impact of the *ccpC* deletion on *L. monocytogenes* virulence factor expression. Furthermore, we examined the impact of *ccpC* deletion on the relative expression of *plcA*, *plcB*, *actA*, and *hly* during the growth of *L. monocytogenes* strains in BHI broth (Figure 1D). The *plcA*, *plcB*, and *actA* genes were significantly downregulated in the Δ*ccpC* strain compared to the wildtype F2365, while the *hly* gene was not significantly different in the Δ*ccpC* strain compared to the wildtype F2365.

### 3.2 CcpC is required for *Listeria monocytogenes* biofilm formation

**Figure 2:**
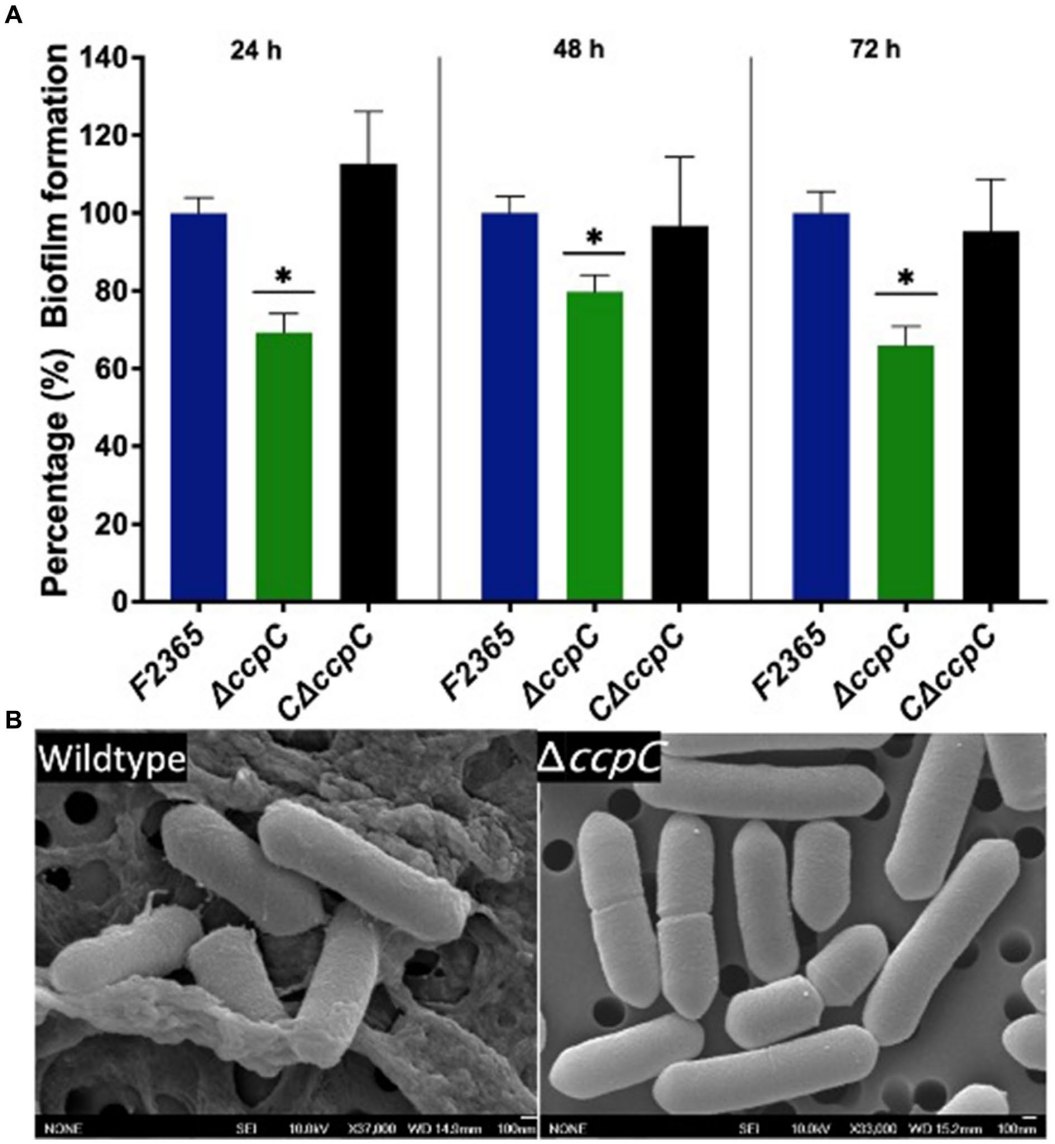
The Δ*ccpC* strain exhibited a reduction in biofilm formation relative to the wildtype F2365 strain. **(A)** Biofilm formation was quantified by measuring the absorbance at 538 nm to detect biofilm crystal violet staining after 24, 48, and 72 h incubations in polystyrene plates. Data represent mean of six replicates ± standard error (SEM). **(B)** Scanning electron microscopy results showing the presence of biofilm formation in wildtype and the absence of biofilm formation for the Δ*ccpC* strain. Data of wildtype F2365 and Δ*ccpC* was compared with Student’s *t*-tests. * Shows significant differences only in the ∆*ccpC* mutant (*p* < 0.05).

The impact of *ccpC* deletion on biofilm formation was determined under static conditions at 24, 48, and 72 h using crystal violet staining. The Δ*ccpC* strain exhibited significantly (*p* < 0.05) reduced biofilm formation compared to F2365 (set to 100%) by approximately 30, 20, and 35% at 24, 48, and 72 h (Figure 2A). Furthermore, scanning electron microscopy revealed that F2365 biofilm exhibited more pronounced complex three-dimensional architecture compared to Δ*ccpC*. Wildtype strain biofilm was characterized by dense microcolonies embedded in a well-defined extracellular polymeric substance (EPS) matrix, unlike the Δ*ccpC* strain, which exhibited individual and scattered bacterial cells with minimal extracellular materials (Figure 2B).

### 3.3 CcpC is not required for efficient intracellular survival of *Listeria monocytogenes*

**Figure 3:**
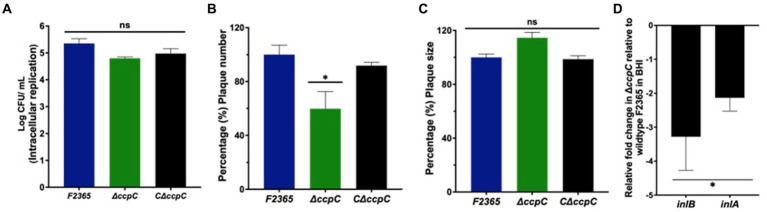
The Δ*ccpC* strain exhibited insignificant intracellular replication in macrophages compared to the wildtype F2365 strain. **(A)** The intracellular replication of the Δ*ccpC* is reduced insignificantly by 0.4 log_10_ compared to F2365 based on CFU recovered from infected macrophages after 5 h (*p* > 0.05). **(B)** Plaque numbers formed by the Δ*ccpC* strain in L2 fibroblasts is approximately 40% reduced compared to the wildtype F2365 strain (*p* < 0.05). The C∆*ccpC* is found to restore the number of plaques to wildtype F2365 level. **(C)** Plaque sizes formed by the Δ*ccpC* were not significantly different when compared to the wildtype F2365 (*p* > 0.05). **(D)** The expression of *inlB* and *inlA* genes during the growth of *L. monocytogenes* strains in BHI broth was significantly downregulated in the Δ*ccpC* strain compared to the wildtype F2365 (*p* < 0.05). The data represent the mean ± SEM. Data were compared with Student’s *t*-tests. Asterisks (*) indicate significant differences, *p* < 0.05. NS, No significant difference.

The ability of Δ*ccpC* to replicate intracellularly in J774 macrophages was reduced by 0.4 log_10_ compared to F2365 based on CFU recovered from infected macrophages after 5 h, but this difference was not significant (*p* > 0.05) (Figure 3A).

Cell to cell spreading of Δ*ccpC* in L2 fibroblasts was significantly decreased compared to F2365 based on plaque diameters and numbers. The Δ*ccpC* strain had 40% reduction in plaque numbers compared to F2365 (Figure 3B). However, plaque size was not significantly affected by the Δ*ccpC* mutation, with a ~ 9% increase relative to wildtype and the CΔ*ccpC* strains (Figure 3C). In order to provide further insights on the reduced plaque number and decipher the impact of *ccpC* deletion on bacterial invasion, we examined the expression of *inlB* and *inlA* genes in the Δ*ccpC* strain. The *inlB* and *inlA* genes were significantly downregulated in the Δ*ccpC* strain compared to the wildtype F2365 during the growth of *L. monocytogenes* strains in BHI broth (Figure 3D).

### 3.4 CcpC contributes to *Listeria monocytogenes* virulence

**Figure 4:**
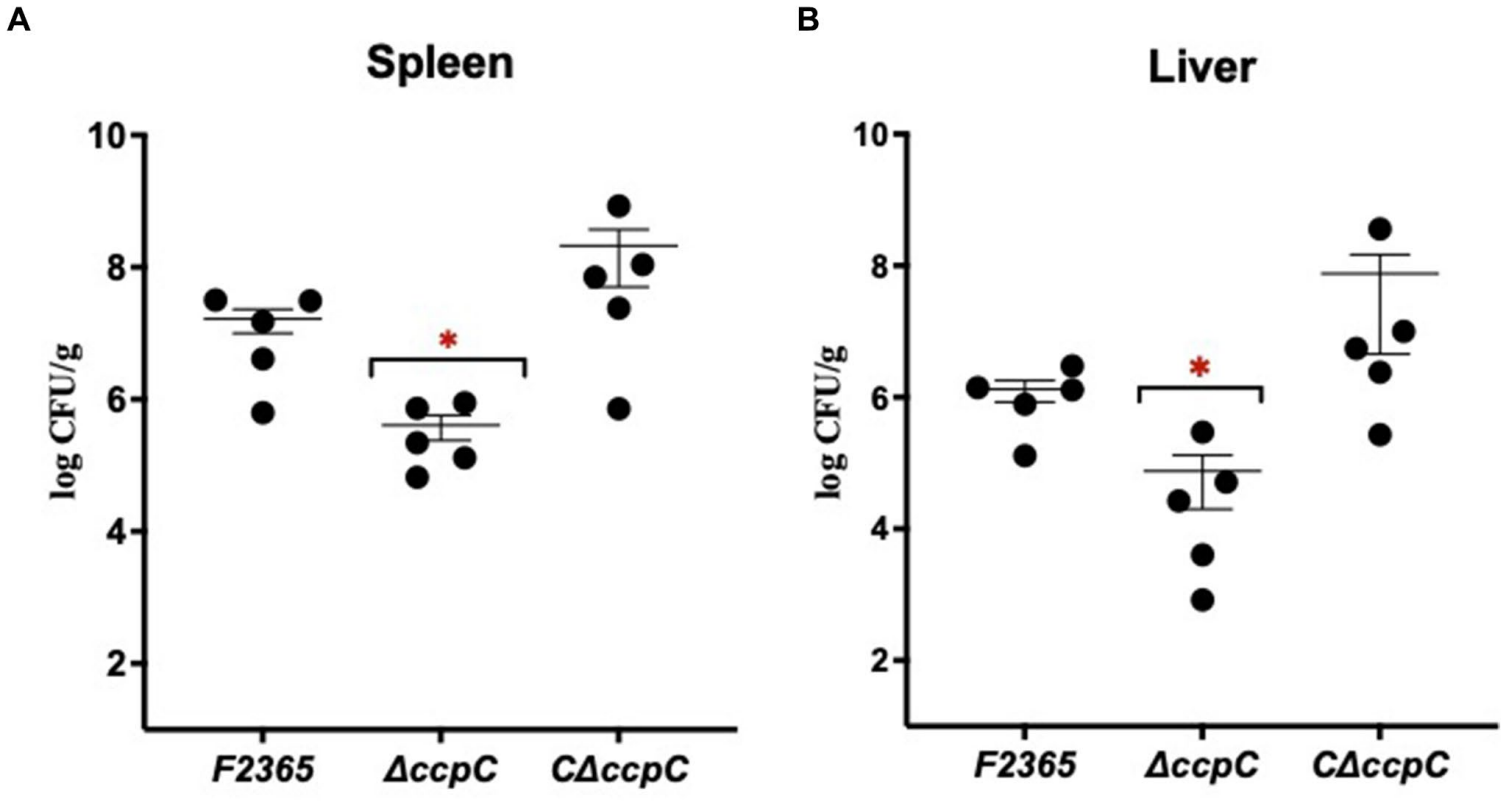
The virulence of ∆*ccpC* bacteria is attenuated in mice. Mice (*n* = 5/cage) were injected with 2 × 10^4^ CFU of the indicated strains. Bacterial burdens were determined for livers **(A)** and spleens **(B)** at 72 h post-infection. Each point represents one mouse. Statistical analysis was performed using a nonparametric Mann–Whitney test. *p < 0.05.

We characterized the virulence of Δ*ccpC* strain using a murine model. At 72 h post-infection, bacterial concentrations in the spleen and liver of mice infected with Δ*ccpC* were significantly lower (2.5 and 1.2 log_10_ CFU reductions, respectively) compared to those infected with F2365 (Figure 4). There were no significant differences in the bacterial concentrations in spleen and liver when comparing mice infected with the C∆*ccpC* and wildtype F2365 strains, indicating that the complemented strain restored the virulence of Δ*ccpC* to wildtype levels.

### 3.5 Deletion of *ccpC* confers better growth and survival to *Listeria monocytogenes* under oxidative stress conditions

**Figure 5:**
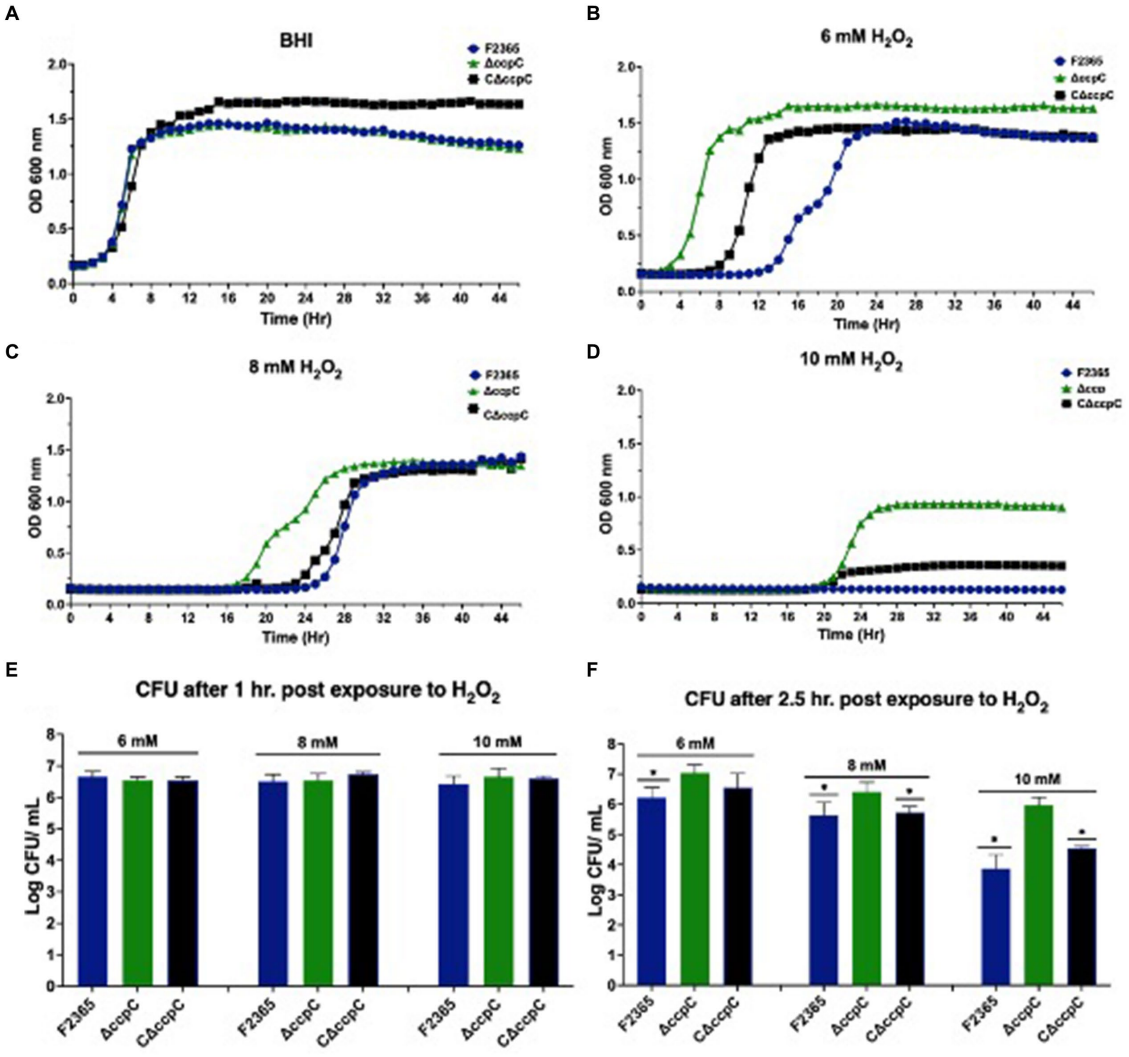
Growth and survival of Δ*ccpC* strain and wildtype F2365 under oxidative stress. **(A)** The Δ*ccpC* strain exhibited a similar growth curve compared to wildtype F2365 and complement strains when grown in BHI at 37°C without oxidative stress. The Δ*ccpC* strain exhibited a decreased lag phase compared to the wildtype F2365 strain when cultured in BHI broth with 6 mM **(B)**, 8 mM **(C)**, and 10 mM **(D)** of H_2_O_2_. Growth assays were performed in a 48-well plate and were repeated at least six times independently with five replicates. Error bars represent the SEM. The survival of the Δ*ccpC* and wildtype F2365 strains was assessed following exposure to 6, 8, or 10 mM H_2_O_2_ for 1 h **(E)** and 2.5 h **(F)**. *L. monocytogenes* strains were grown to the mid-log phase, resuspended in BHI, and treated with H_2_O_2_, after which CFUs were enumerated by colony counts on BHI agar plates. Statistical analysis was performed using Student *t*-test.

When cultured in BHI broth, Δ*ccpC* strain exhibited growth kinetics similar to F2365, implying that *ccpC* is not essential for growth under nutrient-rich conditions (Figure 5A). We further assessed the growth and survival of Δ*ccpC* strain under oxidative stress in BHI containing H_2_O_2_ at concentrations of 6, 8, or 10 mM. Relative to the wildtype strain, the Δ*ccpC* strain exhibited a shorter lag phase (approximately 9 h) compared to F2365 at 6 mM H_2_O_2_ (Figure 5B). At 8 mM H_2_O_2_, the Δ*ccpC* strain exhibited an approximately 9 h difference in the duration of the lag phase as compared to F2365 (Figure 5C). The Δ*ccpC* strain reached the exponential phase faster than wildtype strain. Under high levels of oxidative stress (10 mM H_2_O_2_), the Δ*ccpC* strain showed a longer lag phase duration compared to the lower concentrations of H_2_O_2_, but it entered the exponential phase after approximately 20 h whereas the wildtype strain did not get to the exponential phase even after 48 h (Figure 5D).

We also compared the survival of the wildtype, Δ*ccpC*, and complemented strains after exposure to H_2_O_2_ for 1 and 2.5 h in BHI. In the presence of 6, 8, and 10 mM H_2_O_2_ concentrations, the survival of the Δ*ccpC* strain was higher (0.02, 0.05, and 0.2 log_10_ CFU differences) than F2365 after 1 h, but the differences were not significant (*p* > 0.05) (Figure 5E). After 2.5 h, however, the survival of the Δ*ccpC* strain was significantly higher than that of the wildtype strain under these three H_2_O_2_ exposure levels (0.8, 0.9, and 2.7 log_10_ CFU differences) (Figure 5F). The survival and growth of complemented strain after exposure to H_2_O_2_ was similar to wildtype F2365 strain.

### 3.6 Transcriptomic analyses reveal the extensive impact of CcpC on *Listeria monocytogenes* gene expression

**Figure 6:**
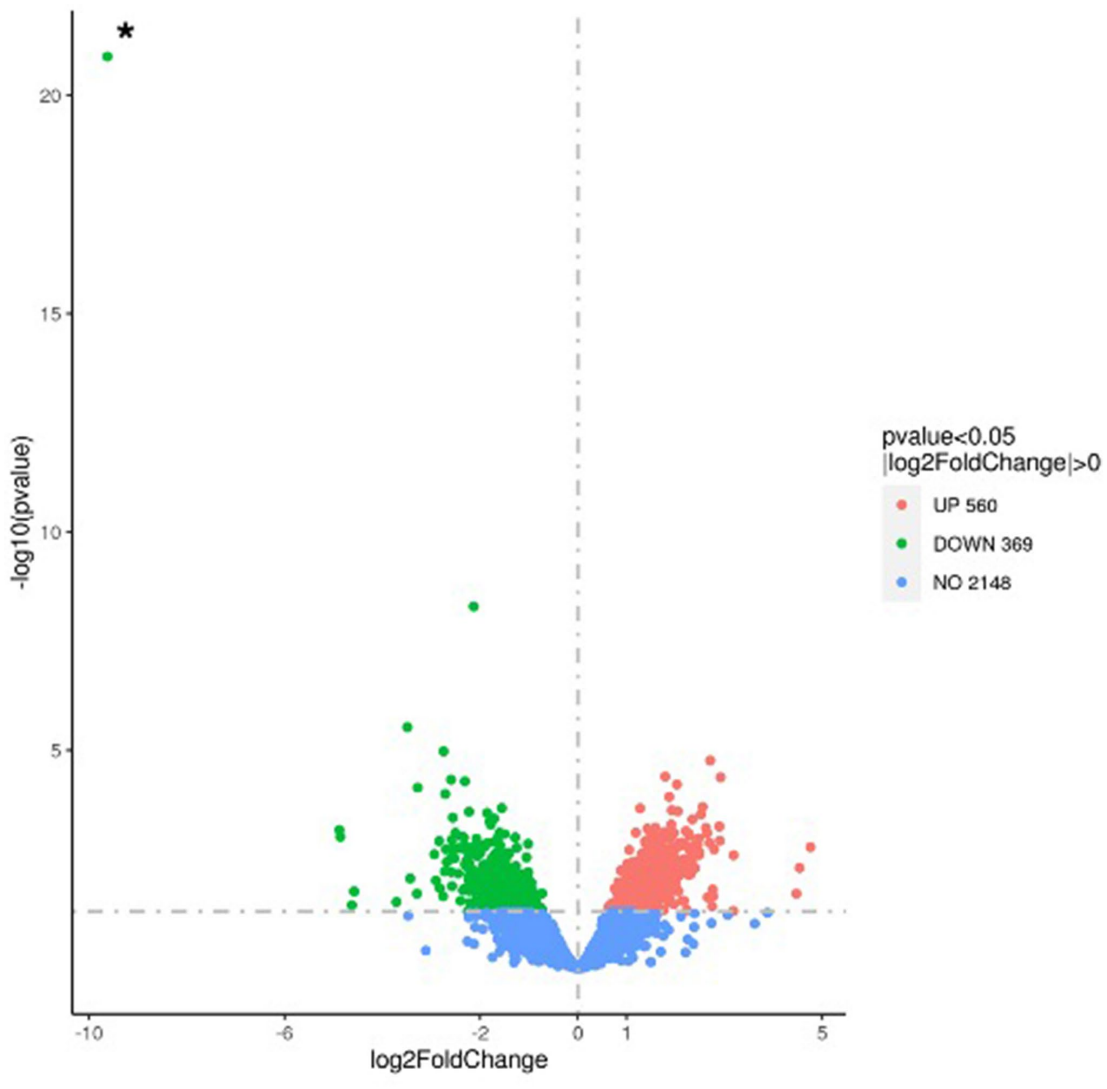
Volcano plot highlighting the differentially expressed genes in the Δ*ccpC* compared to *L. monocytogenes* wildtype F2365 strain following oxidative stress induced by H_2_O_2_. The fold change in expression of each gene is plotted against the significant level (*p*-value) of the corresponding gene expression change. Upregulated genes are plotted in red, and downregulated genes are in green. The asterisk (*) indicates the *ccpC* gene, shown here as the most highly downregulated gene in this comparative analysis.

To further investigate the mechanism underlying the reduced susceptibility of the Δ*ccpC* strain to oxidative stress conditions, we examined the regulatory role of CcpC following exposure to H_2_O_2_-induced oxidative stress. RNA-seq was performed on total RNA harvested from wildtype and Δ*ccpC* cultures that had been grown to exponential phase and then exposed to 8 mM H_2_O_2_ for 2.5 h. Overall, 929 genes were found to be significantly differentially expressed by greater than two-fold (*p* < 0.05), indicating the broad impact of CcpC on *L. monocytogenes* physiology during oxidative stress. Of these differentially expressed genes (DEGs), 560 were upregulated and 369 were downregulated in the Δ*ccpC* strain compared to levels in the wildtype strain (Figure 6). The most upregulated (*n* = 42) and downregulated (*n* = 79) DEGs are summarized in Tables 2, 3.

**Table 2:** The most upregulated genes in the Δ*ccpC* strain compared to the levels in the wildtype F2365 strain.

Gene id	Gene description	log2Fold change	*p* value
**DNA repair**			
LMOf2365_1500	DNA polymerase III, delta subunit	1.4	6.9E-03
LMOf2365_1596	Bacterial DNA polymerase III alpha subunit (*dnaE*)	1.7	3.0E-03
LMOf2365_0177	DNA polymerase III subunit, delta subunit (*holB*)	1.9	3.0E-03
LMOf2365_1587	DNA polymerase I: 5′-3′ exonuclease, C-terminal SAM fold (*polA*)	1.4	6.7E-03
LMOf2365_1417	Protein RecA bacterial DNA recombination protein (*recA*)	2.0	7.9E-04
LMOf2365_0005	DNA replication and repair protein: RecF/RecN/SMC N terminal (*recF*)	1.6	1.4E-02
LMOf2365_1479	DNA repair protein: Recombination protein O N terminal (*recO*)	1.9	2.0E-02
LMOf2365_2682	Recombination protein: Toprim domain (*recR*)	1.8	1.9E-02
LMOf2365_1920	Holliday junction resolvase: Recombination protein U (*recU*)	2.0	5.8E-03
LMOf2365_1552	Holliday junction ATP-dependent DNA helicase: HHH domain (*ruvA*)	1.2	1.3E-02
LMOf2365_1551	Holliday junction ATP-dependent DNA helicase: N-terminus (*ruvB*)	1.3	1.0E-02
LMOf2365_1320	LexA repressor: Peptidase S24-like (*lexA*)	2.0	3.0E-03
LMOf2365_0001	Chromosomal replication initiator protein (*dnaA*)	1.7	4.9E-03
LMOf2365_1924	Replication and repair (*dnaD*)	0.9	4.8E-02
LMOf2365_1586	Formamidopyrimidine-DNA glycosylase HTH domain (*mutM*)	1.5	2.5E-03
LMOf2365_1422	DNA mismatch repair protein: MutS domain V (*mutS*-2)	1.1	1.3E-02
**Iron, zinc and manganese metabolism (PerP system)**			
LMOf2365_1466	Zinc ABC transporter ATP-binding protein ZurA (*zurA*-2)	1.5	3.0E-03
LMOf2365_1464	Transcriptional regulator ZurR (*zurR*)	1.8	2.0E-03
LMOf2365_1707	Peroxide-responsive transcriptional repressor (PerR) (*PerR*)	1.7	7.2E-03
LMOf2365_1986	Ferric uptake regulator family (*fuR*)	1.9	2.2E-03
LMOf2365_1907	Transcriptional regulator MntR: Iron dependent repressor	1.7	9.0E-03
**Protein export**			
LMOf2365_1872	Lipoprotein signal peptidase: Signal peptidase (SPase) II (*lspA*)	2.0	1.0E-02
LMOf2365_0257	Protein translocase subunit (*secE*)	2.1	4.2E-03
LMOf2365_2424	Probable protein-export membrane protein (*secG*)	2.2	1.5E-03
LMOf2365_1287	Signal peptidase IB: Peptidase S24-like (*sipX*)	1.9	2.3E-03
LMOf2365_1288	Signal peptidase I: Peptidase S24-like	1.5	1.7E-02
LMOf2365_1289	Signal peptidase I: Peptidase S24-like	1.6	2.8E-03
**Peptidoglycan biosynthesis**			
LMOf2365_2499	UDP-N-acetylglucosamine 1-carboxyvinyltransferase 1: EPSP synthase (3-phosphoshikimate 1-carboxyvinyltransferase) (*murA*-)1	1.5	1.1E-02
LMOf2365_1439	UDP-N-acetylenolpyruvoylglucosamine reductase, C-terminal domain (*murB*)	1.9	9.0E-04
LMOf2365_1483	Undecaprenol kinas: Prokaryotic diacylglycerol kinase (*dgkA*)	1.5	4.6E-03
LMOf2365_2069	Phospho-N-acetylmuramoyl-pentapeptide-transferase: Glycosyl transferase family 4 (*mraY*)	1.6	1.1E-02
LMOf2365_1332	Isoprenyl transferase: Putative undecaprenyl diphosphate synthase (*uppS*)	1.8	1.2E-02
LMOf2365_0872	D-alanine--D-alanine ligase: C-terminus	1.5	1.5E-02
LMOf2365_2399	Peptidoglycan glycosyltransferase: Cell cycle protein	2.1	1.3E-03
**Teichoic acid biosynthesis**			
LMOf2365_2526	Alpha-galactosylglucosyldiacylglycerol synthase	1.4	2.8E-03
LMOf2365_2527	Alpha-monoglucosyldiacylglycerol synthase: Glycosyl transferases group 1	1.5	1.3E-02
LMOf2365_2491	Uncharacterized Cell envelope-related transcriptional attenuator	1.8	4.2E-03
LMOf2365_1097	Teichoic acid poly(ribitol-phosphate) polymerase: Poly(glycerophosphate) glycerophosphotransferase	1.5	1.0E-02
LMOf2365_0979	Probable undecaprenyl-phosphate N-acetylglucosaminyl 1-phosphate transferase: Glycosyl transferase family 4	1.6	1.8E-02

**Table 3:** The most downregulated genes in the Δ*ccpC* strain compared to the levels in the wildtype F2365 strain.

Gene id	Gene description	log2Fold change	*p* value
**Internal and virulence factor**			
LMOf2365_0282	Internalin-A: Bacterial adhesion/invasion protein N terminal (*inlD*)	−1.5	1.1E-02
LMOf2365_0429	Internalin-A: Bacterial adhesion/invasion protein N terminal (*inlF*)	−1.5	7.9E-03
LMOf2365_0047	Putative agmatine deiminase 1: Porphyromonas-type peptidyl-arginine deiminase	−1.6	1.7E-02
LMOf2365_0281	Internalin-A: Bacterial adhesion/invasion protein N terminal (*inlC2*)	−1.5	5.0E-02
LMOf2365_0289	Internalin-A: Leucine rich repeat	−1.7	2.4E-02
LMOf2365_2418	Internalin B(*inlB*)	−2.6	2.0E-03
LMOf2365_0693	Internalin-I	−1.9	4.3E-03
LMOf2365_1812	Internalin B: Bacterial adhesion/invasion protein N terminal (*inlC*)	−1.8	8.7E-03
LMOf2365_0212	1-phosphatidylinositol phosphodiesterase, X domain (*plcA*)	−2.2	9.1E-03
LMOf2365_0577	Crp/Fnr family transcriptional regulator (*crp/fnr*)	−2.2	9.0E-03
**Propanediol dehydratase (PD) and ethanolamine and porphyrin metabolism**			
LMOf2365_1169	Ethanolamine utilization protein: Cell division protein FtsA(*eutJ*)	−2.0	1.6E-02
LMOf2365_1186	Ethanolamine ammonia-lyase light chain (EutC)	−1.8	3.4E-02
LMOf2365_1187	Ethanolamine utilization protein: BMC domain (*eutL*)	−2.0	3.5E-02
LMOf2365_1162	Propanediol dehydratase medium subunit (*pduD*)	−2.8	1.4E-02
LMOf2365_1173	Ethanolamine utilization protein: Aldehyde dehydrogenase family (*pduP*)	−1.5	1.8E-02
LMOf2365_1154	Bifunctional adenosylcobalamin biosynthesis protein CobU (*cobU*)	−1.5	4.8E-02
LMOf2365_1155	Adenosylcobinamide-GDP ribazoletransferase (*cobS*)	−2.1	3.2E-02
LMOf2365_1200	Cobyrinate a,c-diamide synthase: CobQ/CobB/MinD/ParA nucleotide binding domain (*cobB*)	−2.6	6.2E-03
LMOf2365_1201	Cobalamin biosynthesis protein CobD: CobD/Cbib protein (*cobD*)	−2.2	3.9E-03
LMOf2365_1202	Cobalt-precorrin-8 methylmutase (*cobH*)	−2.7	6.0E-03
LMOf2365_1208	Probable cobalt-factor III C (17)-methyltransferas (*cobJ*)	−2.3	1.5E-03
LMOf2365_1209	Cobalt-precorrin-6A reductase (*cobK*)	−2.4	6.7E-03
LMOf2365_1210	Porphyrin biosynthesis protein: Uroporphyrinogen-III synthase HemD	−2.1	8.2E-03
LMOf2365_1211	Sirohydrochlorin cobaltochelatas: Cobalt chelatase (*cbiK*)	−2.0	2.2E-02
LMOf2365_1212	Cobalt-precorrin-2 C (20)-methyltransferase (*cobI*)	−2.9	2.4E-03
LMOf2365_1213	Cobalt uptake substrate-specific transmembrane region (*cbiM*)	−2.1	2.2E-02
LMOf2365_1214	Cobalt/nickel transport protein cobalt transport protein (*cbiN*)	−2.8	2.2E-02
**Sorbitol/glucitol metabolism**			
LMOf2365_0571	PTS system glucitol/sorbitol-specific EIIA component (*srlB*)	−2.6	1.3E-02
LMOf2365_0572	PTS system glucitol/sorbitol-specific EIIB and C (*srlE*)	−2.0	7.3E-03
LMOf2365_0574	Glucitol operon activator (*gutM*)	−4.6	3.5E-02
LMOf2365_0102	Crp/Fnr family transcriptional regulator	−2.7	1.0E-04
**Phosphotransferase system (PTS)**			
LMOf2365_0024	PTS system fructose-specific EIIA component	−2.7	1.9E-03
LMOf2365_0026	N-acetylgalactosamine permease IIC component	−3.3	7.2E-05
LMOf2365_0044	Glutamine--fructose-6-phosphate aminotransferase	−1.9	4.2E-03
LMOf2365_0113 (*manL*)	PTS system mannose-specific EIIAB component fructose IIA component: PTS system sorbose subfamily IIB component	−1.6	1.0E-03
LMOf2365_0114	PTS system mannose-specific EIIC component	−2.4	9.5E-04
LMOf2365_0115	PTS system mannose-specific EIID component	−2.2	6.9E-03
LMOf2365_0530	Mannitol-specific cryptic phosphotransferase enzyme IIA: EIIA 2	−1.6	1.4E-02
LMOf2365_0532	Phosphoenolpyruvate-dependent sugar phosphotransferase system, EIIA 2	−1.8	3.6E-02
LMOf2365_0659	Transcriptional regulator MtlR: Phosphoenolpyruvate-dependent sugar phosphotransferase system, EIIA 2	−1.8	5.6E-03
LMOf2365_0662	PTS system fructose-like EIIB component 2: PTS system, Lactose/Cellobiose specific IIB subunit	−2.2	1.7E-02
LMOf2365_0892	Ascorbate-specific PTS system EIIA component	−2.1	1.5E-03
LMOf2365_0894	PTS system cellobiose-specific: PTS system, Lactose/Cellobiose specific IIB subunit	−2.3	5.0E-02
LMOf2365_0895	Lichenan permease IIC component	−2.2	2.5E-03
LMOf2365_2167	D-tagatose-1,6-bisphosphate aldolase subunit: Fructose-bisphosphate aldolase class-II	−1.6	4.0E-02
LMOf2365_2168	D-tagatose-1,6-bisphosphate aldolase subunit: Fructose-bisphosphate aldolase class-II	−2.0	8.0E-03
LMOf2365_2622	Ascorbate-specific PTS system EIIB component	−1.9	1.5E-02
LMOf2365_0192	Bacterial extracellular solute-binding protein	−1.8	5.6E-03
LMOf2365_0772	YHCG_BACSU Uncharacterized ABC transporter ATP-binding protein	−2.3	4.2E-03
LMOf2365_1214	Cobalt transport protein	−2.8	2.2E-02
LMOf2365_1754	Putative binding protein (Bacterial extracellular solute-binding protein)	−1.6	1.6E-02
LMOf2365_1756	L-arabinose transport system permease protein	−1.6	1.3E-02
LMOf2365_2032	Probable ABC transporter permease protein	−2.7	3.7E-03
LMOf2365_2157	Maltodextrin transport system permease protein	−2.2	2.3E-03
LMOf2365_2318	L-cystine transport system permease protein	−2.7	1.1E-05
LMOf2365_2553	Putative hemin transport system permease protein: FtsX-like permease family	−1.7	4.3E-03
LMOf2365_2828	Inner membrane ABC transporter permease protein	−1.7	3.2E-02
**Pentose phosphate pathway (PPP)**			
LMOf2365_0363	Fructose-6-phosphate aldolase	−2.1	8.2E-03
LMOf2365_0379	D-tagatose-1,6-bisphosphate aldolase class-II	−1.5	6.2E-03
LMOf2365_0528	Ribulose-phosphate 3 epimerase family	−2.1	9.3E-03
LMOf2365_1054	Putative transketolase C-terminal section	−1.7	1.0E-02
LMOf2365_2167	D-tagatose-1,6-bisphosphate aldolase subunit: Fructose-bisphosphate aldolase class-II	−1.6	4.0E-02
LMOf2365_2168	D-tagatose-1,6-bisphosphate aldolase subunit: Fructose-bisphosphate aldolase class-II	−2.0	8.0E-03
LMOf2365_2641	Ribulose-phosphate 3 epimerase family	−1.8	1.9E-02
LMOf2365_0365 (*rpiB*1)	Ribose-5-P isomerase B	−1.9	1.4E-02
**Amino acids biosynthesis, 2-oxocarboxylic acid metabolism**			
LMOf2365_0363	Probable transaldolase 2: Transaldolase	−2.1	8.2E-03
LMOf2365_0395	Probable triosephosphate isomerase 2 (*tpiA*-1)	−2.0	1.3E-02
LMOf2365_0528	Ribulose-phosphate 3 epimerase family	−2.1	9.3E-03
LMOf2365_1654	Anthranilate synthase component 2: Glutamine amidotransferase class-I (*trpG*)	−1.5	4.9E-02
LMOf2365_1655	Anthranilate synthase component 1, N terminal region (*trpE*)	−1.9	1.6E-03
LMOf2365_2007	Acetolactate synthase large subunit: Thiamine pyrophosphate enzyme, central domain(*ilvB*)	−1.4	3.3E-02
LMOf2365_2008	Acetolactate synthase small subunit: Small subunit of acetolactate synthase (*ilvN*)	−1.9	9.9E-03
LMOf2365_2009	Ketol-acid reductoisomerase [NADP (+): Acetohydroxy acid isomeroreductase, catalytic domain (*ilvC*)]	−1.9	1.5E-02
LMOf2365_2010	2-isopropylmalate synthase (*leuA*)	−2.1	1.1E-03
LMOf2365_2011	3-isopropylmalate dehydrogenase: Isocitrate/isopropylmalate dehydrogenase (*leuB*)	−2.3	1.9E-03
LMOf2365_2012	3-isopropylmalate dehydratase large subunit: Aconitase family (*leuC*)	−2.3	3.9E-03
LMOf2365_2013	3-isopropylmalate dehydratase small subunit: Aconitase C-terminal domain (*leuD*)	−2.2	5.8E-03
LMOf2365_2014	L-threonine dehydratase biosynthetic: Pyridoxal-phosphate dependent enzyme (*ilvA*)	−1.8	2.3E-02

To further identify the significantly enriched metabolic pathways that differed between the Δ*ccpC* and F2365 strains, DEGs were mapped to reference pathways in the KEGG database. Pathways associated with DEGs upregulated in Δ*ccpC* compared to F2365 included homologous recombination, mismatch repair, DNA replication (23 genes), protein export (8 genes), teichoic acid biosynthesis (9 genes), and peptidoglycan biosynthesis (8 genes) pathways (Table 4). Pathways associated with DEGs downregulated in the Δ*ccpC* strain included BCAA biosynthesis (30 genes), PTS (28 genes), fructose and mannose metabolism (15 genes), porphyrin metabolism, 2-oxocarboxylic acid metabolism (7 genes), PPP (8 genes), and selenocompound metabolism pathways (3 genes) (Table 4).

**Table 4:** KEGG pathway enrichment analysis for upregulated and downregulated genes in Δ*ccpC* strain compared to the levels in the wildtype F2365 strain.

Description	*p* value	Corrected *p* value	Gene no.
**Upregulated pathway**			
Homologous recombination	5.72E-05	2.08E-03	23
Terpenoid backbone biosynthesis	6.60E-05	2.08E-03	10
Protein export	5.49E-04	8.65E-03	8
Teichoic acid biosynthesis	1.81E-03	2.28E-02	9
Peptidoglycan biosynthesis	5.47E-03	5.74E-02	8
Nucleotide metabolism	9.63E-02	6.74E-01	10
Glycerophospholipid metabolism	1.19E-01	7.48E-01	5
Two-component system	1.35E-01	7.62E-01	13
Bacterial secretion system	1.76E-01	7.92E-01	4
**Downregulated pathway**			
Phosphotransferase system (PTS)	2.55E-06	1.43E-04	28
Valine, leucine and isoleucine biosynthesis (branched chain amino acids)	3.17E-05	8.89E-04	8
Fructose and mannose metabolism	1.49E-03	2.78E-02	15
Starch and sucrose metabolism	1.13E-02	1.58E-01	16
Inositol phosphate metabolism	1.78E-02	1.87E-01	5
Biosynthesis of amino acids	2.00E-02	1.87E-01	30
Porphyrin metabolism	3.38E-02	2.70E-01	10
2-oxocarboxylic acid metabolism	4.19E-02	2.93E-01	7

To better understand the impact of CcpC on *L. monocytogenes* following H_2_O_2_-induced oxidative stress, gene ontology (GO) analyses were conducted to functionally categorize differentially expressed genes into three broad categories, including biological processes, cellular components, and molecular functions. The most enriched biological process clusters were composed of genes encoding proteins involved in DNA repair, and responses to stress. With respect to cellular component terms, genes associated with the cell periphery, cytoplasmic metabolism, integral membrane component, ribonucleic complex, and ribosome were the most enriched. In the molecular function category, carbon–carbon lyase activity, kinase activity, and transferase activity terms were the most enriched groups (Table 5).

**Table 5:** Gene ontology (GO) enrichment analysis of the differentially expressed upregulated and downregulated genes in Δ*ccpC* compared to the levels in the wildtype F2365 strain.

Category	Description	*p* value	*p*-adj	Count
**GO pathway enrichment analysis for upregulated genes in ΔccpC compared wildtype F2365**				
BP	Nucleic acid metabolic process	5.5E-08	9.0E-06	66
	DNA repair	9.3E-07	3.6E-05	38
	Cellular response to stress	9.3E-07	3.6E-05	15
	Cellular response to stimulus	3.0E-06	8.3E-05	23
	Nucleobase-containing compound metabolic process	1.1E-05	2.3E-04	72
	Heterocycle metabolic process	1.4E-05	2.7E-04	79
	Cellular aromatic compound metabolic process	1.7E-05	2.8E-04	78
	Response to stimulus	1.8E-05	2.8E-04	27
	Organic cyclic compound metabolic process	2.3E-05	3.3E-04	79
	Cellular macromolecule metabolic process	2.5E-05	3.5E-04	63
	Regulation of cellular process	2.0E-04	2.6E-03	43
	Regulation of biological process	2.3E-04	2.8E-03	43
	Cellular nitrogen compound metabolic process	3.4E-04	3.9E-03	81
	Regulation of nucleobase-containing compound metabolic process	4.7E-04	4.4E-03	36
	Regulation of cellular metabolic process	4.7E-04	4.4E-03	36
	Regulation of nitrogen compound metabolic process	4.7E-04	4.4E-03	36
	Regulation of macromolecule metabolic process	4.7E-04	4.4E-03	36
	Regulation of primary metabolic process	5.5E-04	4.4E-03	36
	Biological regulation	5.7E-04	4.4E-03	43
	Regulation of macromolecule biosynthetic process	9.0E-04	6.0E-03	35
**GO pathway enrichment analysis for downregulated genes in ΔccpC compared wildtype F2365**				
BP	Phosphoenolpyruvate-dependent sugar phosphotransferase system	1.0E-06	8.2E-05	66
	Establishment of localization	2.3E-06	8.2E-05	66
	Localization	2.1E-06	8.2E-05	67
	Cobalamin biosynthetic process	1.0E-04	2.5E-03	7
	Vitamin metabolic process	9.7E-04	1.5E-02	9
	Water-soluble vitamin biosynthetic process	9.7E-04	1.5E-02	9
	Tetrapyrrole metabolic process	3.1E-03	4.3E-02	8
MF	Protein-N(PI)-phosphohistidine-sugar phosphotransferase activity	1.7E-05	1.9E-03	16
	Carbohydrate transmembrane transporter activity	4.4E-05	2.5E-03	16
	Phosphotransferase activity, alcohol group as acceptor	9.1E-05	3.4E-03	23
	Active transmembrane transporter activity	2.2E-04	6.3E-03	22
	Transporter activity	6.6E-04	1.5E-02	33
	Carbon–carbon lyase activity	1.3E-03	2.5E-02	7
	Transferase activity, transferring phosphorus-containing groups	4.6E-03	7.4E-02	27
	Lyase activity	6.2E-03	8.8E-02	11
	Transmembrane transporter activity	7.1E-03	8.9E-02	24

GO enrichment analyses revealed that a majority of the genes involved in the cellular response to DNA damage (15 genes), DNA metabolic processes (23 genes), DNA repair (15 genes), cellular responses to stress, and the regulation of nitrogen compound metabolic processes were significantly upregulated in ∆*ccpC* strain relative to the wildtype. In contrast, the main categories represented among downregulated genes were associated with organic substance transport (34 genes), PTS (27 genes), transport localization (66 genes), establishment of localization (67 genes), carbohydrate transport (66 genes), and cobalamin metabolic process (27 genes) (Table 5).

**Figure 7:**
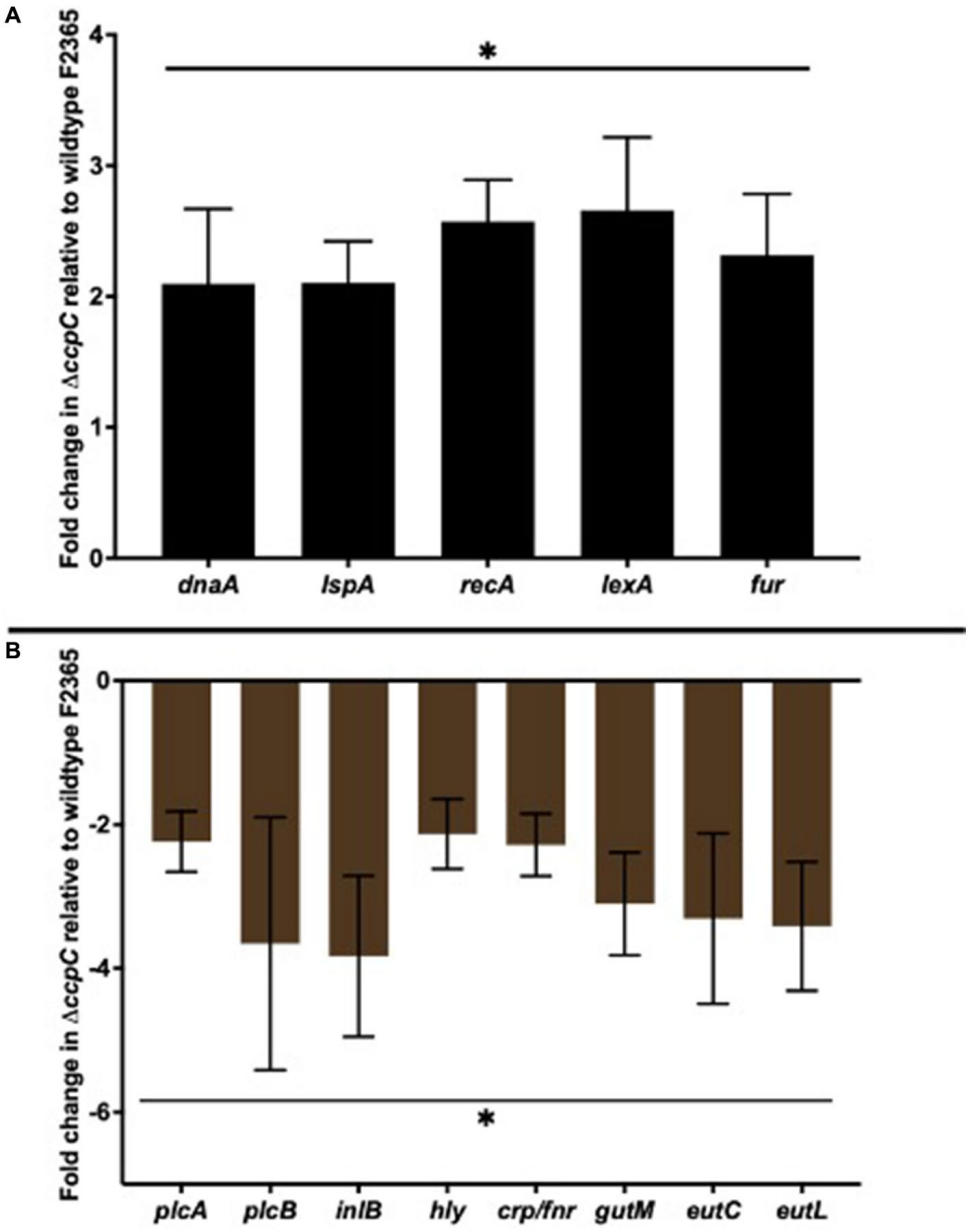
RT-qPCR analyses of select upregulated **(A)** and downregulated **(B)** differentially expressed genes. The selected five upregulated DEGs (*dnaA*, *ispA*, *recA*, *lexA*, and *fuR*) and eight downregulated DEGs (*plcA*, *plcB*, *inlB*, *hly*, *crp/fnr*, *gutM*, and *eutL*) showed expression profiles consistent with the transcriptomic sequencing results. Bars indicate the SEM of the mean of six biological replicates. The data were compared with Student’s *t*-tests. Asterisks (*) indicate significant differences, *p* < 0.05.

Five upregulated DEGs (*dnaA*, *ispA*, *recA*, *lexA*, and *fuR*) and eight downregulated DEGs (*plcA*, *plcB*, *inlB*, *hly*, *crp/fnr*, *gutM*, and *eutL*) were selected to validate their expression by RT-qPCR. The 16S rRNA housekeeping gene was used for normalization. The expression profile of the upregulated and downregulated genes was consistent with the transcriptomic sequencing results (Figure 7).

## 4 Discussion

This study aimed to provide new insight regarding the contribution of LysR-type transcriptional regulator (LTTR) CcpC to the pathogenesis, physiology, and stress responses of *L. monocytogenes* while also seeking to clarify the relationship between CcpC and other virulence factors. Given that a number of gene products directly regulated by PrfA are known to contribute to *L. monocytogenes* virulence, we evaluated the LLO-mediated hemolytic activity and phospholipase activities (mediated by PlcA and PlcB) of the Δ*ccpC* strain. *L. monocytogenes* uses phospholipase and LLO to mediate vacuolar escape into host cell cytoplasm and to achieve cell-to-cell spread (Freitag et al., 2009; Kanki et al., 2018).

Here, we found that the deletion of *ccpC* slightly reduced hemolysis to ~98% of wildtype levels, while reducing the amount of LLO protein to ~75% of wildtype levels and that of phospholipase activity to ~80%. It is possible that other factors contribute to the reduction of LLO secretion in the Δ*ccpC* strain, as there is no correlation observed between LLO protein levels and hemolytic activity. It is also reasonable to believe that a 25% reduction in LLO level is insufficient to yield a low hemolysis level. The expression level of *hly* was not significantly changed in the Δ*ccpC* strain compared to wildtype, providing evidence that deletion of *ccpC* not affect the hemolytic activity of *L. monocytogenes* toward sheep erythrocytes. Although there have been limited reports linking CcpC to hemolysis and LLO expression in *L. monocytogenes*. This finding suggests that CcpC may be involved directly or indirectly in the secretion and activity of LLO and phospholipases (Mitchell et al., 2018). However, further insights is needed on the impacts of CcpC on the secretion of these virulence proteins.

We found that deletion of *ccpC* reduced the ability of *L. monocytogenes* to form biofilms. Despite the paucity of information regarding the contribution of CcpC to biofilm formation in *L. monocytogenes*, many LTTRs (PrhO, BvlR, VtlR, LeuO, and BvlR0) contribute to biofilm formation in *R. solanacearum*, *P. aeruginosa*, *Agrobacterium tumefaciens*, *E. coli*, *Salmonella enterica*, *V. cholerae*, and *Yersinia enterocolitica* (Shimada et al., 2011; McCarthy et al., 2014; Zhang et al., 2018; Budnick et al., 2020; Islam et al., 2021). Biofilms are important for the persistence of *L. monocytogenes* on many different surfaces (Nowak et al., 2021). Therefore, it is possible that CcpC plays a complex regulatory role in *L*. *monocytogenes*, and its functions appear to include several cellular processes.

Interestingly, the Δ*ccpC* strain displayed no significant defects in intracellular growth in macrophages and exhibited normal sized plaques, indicating that the impact of CcpC on the cell to cell spread by *L. monocytogenes* is limited. This finding suggests that CcpC does not play a major role as a regulator of intracellular replication in tissue culture despite the observed reductions in LLO levels and phospholipase activity. Modest reductions in secreted LLO activity have generally not been linked with significant intracellular growth defects, as bacterial strains that exhibit ~25% LLO activity in comparison to wildtype strains still display normal patterns of vacuole escape and intracellular growth (Xayarath and Freitag, 2012; Phelps et al., 2018). The significant reduction in plaque numbers and expression of *inlB* and *inlA*, which could indicate that *L. monocytogenes* has reduced invasive and adhesion abilities after *ccpC* deletion.

In this study, the Δ*ccpC* strain exhibited decreased bacterial concentrations in the liver and spleen of Swiss Webster mice. This result strongly supports a role for CcpC in *L*. *monocytogenes* pathogenesis. LTTRs have been associated with virulence in other bacterial species, such as MetR, GlyA1, MetJ, and LeuO in *E. coli*, PrhO in *Ralstonia solanacearum*, MvfR in *Pseudomonas aeruginosa*, and ShvR in *B. cenocepacia* (Bernier et al., 2008; Bogard et al., 2012; Aktories et al., 2016; Zhang et al., 2018). Together, this data suggests that the *in vivo* virulence defect observed in the Δ*ccpC* strain occurs through a mechanism other than a defect in intracellular replication.

Surprisingly, our results indicated that deletion of *ccpC* increased the ability of *L. monocytogenes* to tolerate H_2_O_2_, indicating that CcpC regulates oxidative stress response. Oxidative stress-related damage is a potent bactericidal mechanism by which professional phagocytes can limit the systemic spread of *L. monocytogenes* (Flannagan et al., 2015; Herb and Schramm, 2021). Previous studies have revealed that the deletion of the LTTR *oxyR* in *N. gonorrhoeae* resulted in a strain that was highly resistant to H_2_O_2_-induced stress. It is noteworthy that *ccpC* deletion improved stress response under the H_2_O_2_-induced stress, however wildtype F2365 may be able to respond to oxidative stress just as well as the *∆ccpC* strain under other conditions, including during intracellular growth in macrophages.

To investigate the role of CcpC in *L*. *monocytogenes* under conditions of oxidative stress, we identified the genes controlled by CcpC via RNA-seq. Sixteen integral genes involved with DNA repair machinery were upregulated in the *∆ccpC* strain compared to F2365 after exposure to H_2_O_2_-induced oxidative stress. These genes include DNA recombination proteins (*recU*, *recA*, *recF*, *recR*, and *recO*), holliday junction ATP-dependent DNA helicase (*ruvA*, *ruvB*), *lexA* repressor, replication and repair (*dnaA*, *dnaD*, and *dnaE*), DNA polymerase (*polA* and *holB*), and DNA mismatch repair proteins (*mutM* and *mutS*). These genes are involved in various processing steps of DNA replication and play roles in the restarting of stalled replication forks, homologous recombination, the repair of double-stranded DNA breaks, and the introduction of adaptive point mutations (Koroleva et al., 2007; Henry and Henrikus, 2021; Matsuda et al., 2022). These results indicate that the increased expression of DNA damage repair proteins in the absence of CcpC regulation improves the response of ∆*ccpC* bacteria to oxidative stress-induced DNA damage.

Previous studies have shown that bacterial responses to DNA damage are mediated by the SOS response pathway, which promotes the repair and survival of DNA-damaged bacteria and the induction of genetic variation in stressed and stationary-phase bacteria (Matsuda et al., 2022). In *L. monocytogenes*, the SOS response pathway is regulated by LexA and RecA (Henry and Henrikus, 2021), and expression of *L*. *monocytogenes* recombination proteins is elevated in response to DNA-damaging agents (Ojha and Patil, 2020). Indeed, studies of stress-related survival have shown that a Δ*recA L. monocytogenes* mutant is less resistant to heat, H_2_O_2_, and acid exposure relative to wild-type strain. Overall, this finding clearly demonstrates the importance of the *ccpC* gene for the ability of *L. monocytogenes* to adapt to oxidative stress by significantly contributing to various forms of SOS response pathway activity such as DNA repair and DNA stability.

In addition to effects on DNA repair proteins, the deletion of *ccpC* increased the transcription of five metalloregulatory proteins, including ferric uptake regulator family (*fur*), the peroxide-responsive transcriptional repressor PerR (*perR*), zinc ABC transporter ATP-binding protein (*zurA*), a zinc transcriptional regulator (*zurR*), and the transcriptional regulator MntR. In many Gram-positive bacteria, Fur regulates iron uptake and siderophore biosynthesis, Zur regulates two ABC zinc transporters, and PerR regulates the oxidative stress response (Horsburgh et al., 2001; Zhang et al., 2012; Pi and Helmann, 2017). Deletion of *perR* causes enhanced resistance to H_2_O_2_ in *B. subtilis* (Zhang et al., 2012), *C. acetobutylicum* (Hillmann et al., 2008), *S. aureus* (Horsburgh et al., 2001), *S. pyogenes* (Brenot et al., 2005), and *Streptococcus suis* (Zhang et al., 2012). The upregulation of these metalloregulatory genes (*perR*, *fuR*, *zurR*, and *zurA*) directly supports the observed increased resistance and survival of *∆ccpC* strain under conditions of H_2_O_2_-induced stress. It is also possible that CcpC plays an important role in metal ion homeostasis in *L. monocytogenes* by regulating zinc and iron uptake genes. Together, these findings indicate that *L. monocytogenes* reacts to oxidative stress by downregulating expression of Fur/PerR-regulated genes involved in iron/zinc uptake and utilization through CcpC.

In this study, several genes involved in different routes of protein export were found to be upregulated in *∆ccpC* following H_2_O_2_-induced oxidative stress, including genes encoding secretomes (*secE* and *secG*), ATP-dependent Clp endopeptidase (*clp*), Type-I signal peptidases (*sipX*, *sipY*, and *sipZ*), and lipoprotein signal peptidase (*lspA*). These pathways are likely used for the export of certain virulence factors to the bacterial surface in response to changes in the environment. It is likely that signal peptidases and secretory proteins allow *L. monocytogenes* to deal with oxidative stress following H_2_O_2_ exposure by increasing its capacity to export certain proteins, reacting in parallel to prevent further uptake of H_2_O_2_.

Genes involved in the biosynthesis of peptidoglycan, teichoic acids, and cell wall proteins were also upregulated in ∆*ccpC* as compared to F2365 following H_2_O_2_-induced oxidative stress. Both peptidoglycan and teichoic acid biosynthesis stem from the precursor molecule UDP-N-acetyl-α-D-glucosamine (UDP-GlcNAc). These findings suggest an increased rate of peptidoglycan and cell envelope turnover following H_2_O_2_ exposure. This increased turnover rate may reflect the increased growth rate and survival of the ∆*ccpC* strain.

Transcriptomic analysis revealed repression of genes encoding internalins and 1-phosphatidylinositol phosphodiesterase in *∆ccpC* compared to the wildtype F2365 strain. PrfA regulates internalins (*inlA* and *inlB*) and *plcA*, which are critical for *L. monocytogenes* pathogenicity. InlA and InlB are important for cell invasion, the induction of bacterial uptake into the nonphagocytic/epithelial cells, and traversal of the intestine–blood barrier. Internalin proteins all share a leucine-rich-repeat domain that allows them to bind to structurally unrelated ligands, thereby implicating them in a wide range of functions (Kobe and Kajava, 2001).

Another important finding of this study was decreased expression of propanediol dehydratase (PD) utilization genes, ethanolamine (EA) pathway genes, and cobalamin biosynthesis genes in the *ccpC* mutant relative to F2365 under oxidative stress. Enzymes required for the metabolism of PD and EA are dependent on cobalamin derivatives as cofactors. *L. monocytogenes* uses PD and EA to maintain the bacterial microcompartment (Joseph and Goebel, 2007; Chowdhury et al., 2014; Anast et al., 2020). EA is used by *L. monocytogenes* as an alternative to nitrogen source (Kutzner et al., 2016; Kaval and Garsin, 2018). The *pdu* and *eut* genes are important for *L. monocytogenes* pathogenicity, and increased expression of these genes has been reported in the gastrointestinal tract and blood of mice (Kaval and Garsin, 2018; Anast et al., 2020). Several transcriptomic studies have shown the upregulation of EA and PD metabolism genes and cobalamin biosynthesis genes under a variety of food and food production environment-related stress conditions (Hingston et al., 2017). This finding indicates that *L. monocytogenes* employs CcpC to promote the expression of these genes during oxidative stress.

Several genes that play a role in the sorbitol/glucitol transport and metabolism exhibited reduced expression in the ∆*ccpC* strain as compared to the wildtype F2365 strain, including glucitol operon activator (*gutM*), glucitol/sorbitol-specific EIIA component (*srlB*), glucitol/sorbitol-specific EIIB and C (*srlE*), LMOf265_*0573*, SAF domain-containing protein, sugar-binding transcriptional regulator, and Crp/Fnr family transcriptional regulator. The operon is involved in the transport and phosphorylation of sorbitol to sorbitol-6-phosphate and the conversion of sorbitol-6-phosphate to fructose-6-phosphate with the associated reduction of NAD^+^ to NADH (Boyd et al., 2000; Murinda et al., 2004). The downregulation of the genes in the *gut* operon would translate to a reduced nutrient pool and subsequent energy deprivation owing to *ccpC* deletion in *L. monocytogenes*. This result supports a role for CcpC as a positive regulator of the expression of this operon in *L. monocytogenes* wildtype strain.

In this study, 27 PTS genes were downregulated in *∆ccpC* strain as compared to the wildtype F2365 strain. The PTS is an integral system for sugar transportation and phosphorylation through three to four protein domains termed IIA, IIB, IIC, and IID (Jeckelmann and Erni, 2019). PTSs utilize phosphate to facilitate the uptake of simple sugars and thus consume more energy than other membrane kinases with the same sugar specificity (Mengaud et al., 1991). This suggests that *∆ccpC* cells may benefit from employing alternative sugar uptake systems and downregulating sugar uptake when exposed to oxidative stress to conserve energy for more critical ROS defense mechanisms.

Intriguingly, some of the genes associated with the PPP were downregulated in the *∆ccpC* strain relative to wildtype strain, including genes encoding including fructose-6-phosphate aldolase, D-tagatose-1,6-bisphosphate aldolase, ribulose-phosphate 3-epimerase, transketolase, C-terminal domain, probable transaldolase, and ribose/galactose isomerase. The PPP is composed of two branches, an oxidative and a non-oxidative branch. Glucose flux through the oxidative branch produces NADPH, an essential reducing agent involved in detoxification and the protection of bacteria from ROS (Dons et al., 2014; Cheng et al., 2017; Abdelhamed et al., 2022). The non-oxidative branch generates the five-carbon sugar from glucose. Previous studies have reported that the upregulation of oxidative PPP is particularly important for supplying NADPH during acute oxidative stress. Therefore, our findings may indicate that *L. monocytogenes* may benefit from upregulation of PPP components during oxidative stress. This finding highlights CcpC as a key factor that regulates *L. monocytogenes* physiology and responses to diverse stressors by controlling the expression of important metabolic pathways.

In this study, genes encoding enzymes involved in the BCAA biosynthesis operon were downregulated in the *∆ccpC* strain as compared to the wildtype F2365 strain. BCAAs are integral for the nutritional requirements of *L. monocytogenes* (Kang et al., 2019). Previous studies have noted a link between BCAAs and virulence gene expression through CodY, which positively regulates PrfA expression in response to low BCAA levels (Keeney et al., 2009). This finding suggests a direct and/or indirect role for CcpC in modulating amino acid metabolism and the expression of the *ilv-leu* operon under stressful conditions. We thus speculate that the downregulation of BCAA biosynthesis may be a consequence of the adaptation and increased resistance of ∆*ccpC* bacteria to oxidative stress.

The *ccpC* gene is flanked upstream by *cbpB* gene, which encodes a c-di-AMP binding protein that acts as a homeostatic regulator of cellular concentrations of (p)ppGpp in response to reduced c-di-AMP levels by regulating the enzymatic functions of RelA (Peterson et al., 2020). In the present study, deletion of *ccpC* had no effect on expression of *cbpB* under H_2_O_2_-induced oxidative stress. However, we could not exclude interactions between *ccpC* and *cbpB* under other environmental conditions, like growth in nutrient rich mediums or in in different nitrogen sources. Interestingly, genes encoding enzymes involved in the TCA (*citZ*, *citB*, and *citC*) were not differentially expressed in the *∆ccpC* strain relative to the wildtype strain in response to H_2_O_2_ treament, suggesting that the TCA metabolites and citric acid play a limited role in the *L. monocytogenes* response to oxidative stress.

These data, together with previous results, suggest that CcpC is likely to be involved in the fine-tuning of the expression of genes involved in stress responses, metabolic activity, and virulence. Bacterial species possess a diverse range of defense mechanisms for sensing, avoiding, and removing oxidants. We herein demonstrated that the induction of DNA repair machinery, SOS responses, and the PerR system in *L*. *monocytogenes* allows better survival under H_2_O_2_-induced oxidative stress. This study provides insights into the mechanisms that govern oxidative stress defenses in *L. monocytogenes*, which may aid in the future development of treatments and preventative strategies for diseases caused by these bacteria. In response to the food processing plant environment, *L. monocytogenes* appear to have developed a variety of defense mechanisms to protect itself against an oxidative environment. Furthermore, research findings from studies of H_2_O_2_ can be directly applicable to bacterial damage and death caused by chemicals or radiation that generate of either free radical species or reactive oxygen species.

## Data availability statement

The data presented in this study are deposited in the NCBI GEO repository with accession number GSE267669.

## Ethics statement

The animal study was approved by the Institutional Animal Care and Use Committee (IACUC) for animal procedures (18-508), and experiments were conducted at the College of Veterinary Medicine. The study was conducted in accordance with the local legislation and institutional requirements.

## Author contributions

SO: Writing – original draft, Software, Methodology, Data curation, Formal analysis. SI: Writing – review & editing, Methodology, Formal analysis. QC: Writing – review & editing, Methodology. OO: Writing – review & editing, Methodology. ML: Validation, Writing – review & editing. HA: Visualization, Supervision, Resources, Investigation, Funding acquisition, Formal analysis, Writing – review & editing.
